# Integrative Chemical–Biological Grouping of Complex High Production Volume Substances from Lower Olefin Manufacturing Streams

**DOI:** 10.3390/toxics11070586

**Published:** 2023-07-05

**Authors:** Alexandra C. Cordova, William D. Klaren, Lucie C. Ford, Fabian A. Grimm, Erin S. Baker, Yi-Hui Zhou, Fred A. Wright, Ivan Rusyn

**Affiliations:** 1 Interdisciplinary Faculty of Toxicology, School of Veterinary Medicine and Biomedical Sciences, Texas A&M University, College Station, TX 77843, USA; 2 Department of Veterinary Physiology and Pharmacology, School of Veterinary Medicine and Biomedical Sciences, Texas A&M University, College Station, TX 77843, USA; 3 Department of Chemistry, University of North Carolina at Chapel Hill, Chapel Hill, NC 27599, USA; 4 Departments of Statistics and Biological Sciences and Bioinformatics Research Center, North Carolina State University, Raleigh, NC 27606, USA

**Keywords:** UVCB, petroleum, regulatory risk assessment, read-across, ion mobility spectrometry

## Abstract

Human cell-based test methods can be used to evaluate potential hazards of mixtures and products of petroleum refining (“unknown or variable composition, complex reaction products, or biological materials” substances, UVCBs). Analyses of bioactivity and detailed chemical characterization of petroleum UVCBs were used separately for grouping these substances; a combination of the approaches has not been undertaken. Therefore, we used a case example of representative high production volume categories of petroleum UVCBs, 25 lower olefin substances from low benzene naphtha and resin oils categories, to determine whether existing manufacturing-based category grouping can be supported. We collected two types of data: nontarget ion mobility spectrometry-mass spectrometry of both neat substances and their organic extracts and in vitro bioactivity of the organic extracts in five human cell types: umbilical vein endothelial cells and induced pluripotent stem cell-derived hepatocytes, endothelial cells, neurons, and cardiomyocytes. We found that while similarity in composition and bioactivity can be observed for some substances, existing categories are largely heterogeneous. Strong relationships between composition and bioactivity were observed, and individual constituents that determine these associations were identified. Overall, this study showed a promising approach that combines chemical composition and bioactivity data to better characterize the variability within manufacturing categories of petroleum UVCBs.

## 1. Introduction

Regulatory agencies commonly categorize chemicals by the amount that is produced and/or imported into a particular jurisdiction; for example, substances whose aggregate quantities exceed some predefined amount per year are considered “high production volumes.” In the European Union, this would entail >1000 tons, and in the United States, the typical cutoff is >1 million pounds (~500 tons). Such substances receive heightened attention in terms of their hazard and risk evaluations and are typically subject to the most extensive testing requirements [1,2]. While most high-production-volume substances are mono-constituent chemicals, a large proportion are derivatives of petroleum refining that belong to a broad class of “unknown or variable composition, complex reaction products, or biological materials” substances (UVCBs). UVCBs from petroleum refining streams pose unique challenges to regulators, both for registration and for human/ecological safety assessments [3,4,5]. These UVCBs are produced from crude oil, which is itself a highly complex and variable material; further, these substances are manufactured not to have an exact composition, but to meet technical specifications related to their use [6].

For regulatory registration and safety evaluation, petroleum UVCBs are grouped into categories of similar materials based on rather broad considerations about their composition and manufacturing methods [7]. It is assumed that substances manufactured to similar performance characteristics will have similar toxicological properties. These assumptions were the basis for the industry’s voluntary data submissions on the mammalian toxicological hazards of petroleum UVCBs under the US EPA High Production Volume (HPV) Challenge Program in the early 2000s [8]. This program established broad categories of petroleum UVCBs based on physio-chemical properties and refining parameters, such as similar boiling ranges, process histories, or end-use types. However, regulators have been less than satisfied with this approach, especially in Europe, and have invited more informed justifications, such as detailed information on constituents, the extent of compositional variability, and assurances that the material that has been (or will be) used for any additional experiments is representative [9,10,11,12,13,14].

To overcome the challenges of grouping and read-across of petroleum UVCBs, two approaches have been recently proposed and tested. In the first approach, human cell-based in vitro studies have been conducted on a large number of substances and categories. These studies tested the hypothesis that in vitro biological activity signatures, both phenotypic and gene expression, can be used to support the grouping of UVCBs [15]. As many as 141 petroleum substances from 16 manufacturing categories [6] were tested in a compendium of 15 human cell types representing a variety of tissues [16]; of these, 6 cell types were also profiled for gene expression [17]. Petroleum substances were assayed in dilution series to derive point of departure (POD) estimates for bioactivity in each phenotype. While it was found that bioactivity was strongly correlated with the content of polycyclic aromatic compounds (PAC), the analysis also revealed substantial variability in bioactivity within each category. Some of these data were used in regulatory submissions to request waivers of animal testing requirements. However, the European Chemicals Agency (ECHA) did not accept the data as presented, in part because of the lack of detailed chemical compositional information [10].

Indeed, efforts to provide more detailed compositional characterization constitute a second approach to refining the current read-across of petroleum UVCBs. While there are many analytical methods that have been used to characterize the composition of these substances [7], they are largely insufficient for meeting regulatory requirements [14]. A number of novel ultra-high resolution and multi-dimensional mass spectrometry-based methods have been applied for the analysis of petroleum samples; however, most of these are yet to be adopted by industry or used in regulatory submissions [5]. Further, ultrahigh-resolution instruments and computational methods enabled the confident determination of molecular formulae for a large portion of these constituents in petroleum UVCBs [18,19]. The advantages of these novel techniques, such as ion mobility spectrometry-mass spectrometry (IMS-MS), as complements to traditional gas chromatography-mass spectrometry (GC-MS) have been demonstrated in a number of regulatory contexts—for grouping of crude oils [20] and petroleum UVCBs [21,22], for chemical speciation of oil weathering by-products [23,24], and for characterization of compositional variability of petroleum UVCBs [25].

While both bioactivity and detailed chemical analyses have been used separately to evaluate similarity in petroleum UVCBs, a combination of the approaches has not been undertaken. Inclusion of PAC and other physio-chemical properties together with cell-based bioactivity did show advantages to data interpretation [15,16,17]; therefore, an investigation of the utility of high-resolution analytical data is also warranted. Herein, a case example of representative high production volume categories of petroleum UVCBs, two lower olefin manufacturing streams, was used to determine whether the existing grouping of the individual substances into these categories and further into “human health hazard” subcategories as defined under the US EPA HPV Challenge Program can be supported by the data from the new approach methodologies that included probing of both bioactivity and chemical composition. We tested 25 lower olefin substances belonging to the low benzene naphthas (LBN) and Resin Oils and Cyclodiene Dimer Concentrates (RO) categories. We collected two types of data: nontarget high-resolution IMS-MS analyses of each neat substance and their respective dimethyl sulfoxide (DMSO) extract, along with in vitro bioactivity of the DMSO extracts in five different cell types: human umbilical vein endothelial cells (HUVEC), as well as induced pluripotent stem cell (iPSC)-derived hepatocytes, endothelial cells, neurons, and cardiomyocytes. Using these data, we grouped substances and compared the groupings to those in the classes/sub-classes established by the HPV Challenge Program.

## 2. Experimental Section

### 2.1. Substances Used in This Study

All lower olefin substances used in this study (assigned number identifiers) and their respective streams are detailed in Table 1. In total, 25 neat substances (identified as 13 resin oils and 12 low benzene naphthas) were included in the analyses and were donated by member companies of the American Chemistry Council’s (ACC) Olefins Panel. Both the identity and origin of the individual substances were de-identified beyond each substance’s Chemical Abstract Service (CAS) number and manufacturing stream name. Select samples were categorized into different human health subcategories than originally proposed under the HPV Challenge Program based on the expert judgement of the authors and the information provided by the manufacturers. Table S1 details our reasoning for group assignments.

Samples were stored at −80 °C until analyzed or otherwise processed. From each substance, an organic extract was prepared using DMSO and cyclohexane, a method that preferentially extracts PAC from petroleum-containing samples, according to the standard American Society for Testing and Materials (ASTM) IP 346 method [28]. Briefly, 4 g of each substance was dissolved in 10 mL cyclohexane. The cyclohexane fraction was then extracted twice with 10 mL pre-equilibrated 10:1 DMSO/cyclohexane. The two subsequent DMSO fractions were collected in a 20 mL glass vial and stored at −80 °C until used in the experiments. It is important to note that throughout this study, the substances are referred to by a five-digit ID (e.g., 84070) prefixed by either “N” representing a “neat” substance, or “E” representing its DMSO extract.

### 2.2. IMS-MS Analysis of Neat Substances and DMSO Extracts

All substances were analyzed using an ion mobility spectrometry (IMS) instrument coupled to a quadrupole time-of-flight (QTOF) mass spectrometer (MS) (model G6560A, Agilent Technologies, Santa Clara, CA, USA). Neat and extracted samples were prepared for IMS-MS analysis as follows. A glass syringe was first used to add 100 µL of each sample to a glass vial. Substances were then diluted 3× by adding 200 µL of 50:50 acetonitrile/toluene buffer and vortexing. The glass syringe was rinsed in triplicate with acetone, hexane, and methanol between the preparation of each sample. All samples were analyzed using an atmospheric pressure photoionization (APPI) source in positive ion mode and were injected at a flow rate of 50 µL/min. The appropriate tune mix was used to calibrate the instrument prior to sample runs, and samples were collected for 1.5 min each. Washes with acetone and methanol were conducted at least three times between samples. Other instrument parameters were consistent with prior studies examining similar substances using an APPI ion source in positive mode [29].

Upon acquisition, IMS-MS raw data files for neat substances and corresponding extracts were first calibrated in IMS-MS Browser B.08.00 software (Agilent Technologies, Santa Clara, CA, USA) using the tune mix file obtained prior to the sample run. The tune mix file was verified to have mass accuracies within ±5 ppm *m*/*z* for each calibrant peak. Calibrated files for neat substances and extracts were then processed using Agilent Mass Profiler software to obtain two separate sets of detected compounds, or “features”, and their abundances in each sample. A library of compounds was then used to match identities to detected features based on *m*/*z* and collisional cross section (^DT^CCS_N2_) values for each compound [30]. ^DT^CCS_N2_ values are a quantitative representation of the size and shape of individual features, derived from the drift time (DT) of each feature [31,32,33]. ^DT^CCS_N2_ is unique to each detected species and can be used to identify targeted species within a nontarget dataset [18,32]. Datasets for neat substances and extracts, including library-matched anchor features, were then exported from MassProfiler for chemical characterization. Raw IMS-MS data files for neat samples and extracts can be found in Tables S2 and S3, respectively.

Chemical characterization was conducted following a modified workflow detailed previously [18]. In brief, datasets were first processed to only include features at an abundance ≥ 5000 in at least one sample to minimize unnecessary amplification of noise. Filtered data matrices can be found in Tables S4 and S5 for neat samples and extracts, respectively. Anchor features were then manually verified using the ^DT^CCS_N2_ library to ensure *m*/*z* fell within a range of ±5 ppm and ±mDa and ^DT^CCS_N2_ values fell within a range of ±1%. Kendrick mass defect (KMD) was then calculated in the context of CH_2_ functional units to enable feature organization in homologous series and molecular formula identification of hydrocarbon species. The series were then validated using KMD-H homologous series and ^DT^CCS_N2_ values [18]. Once a maximum number of features were characterized with confidence, double bond equivalence (DBE) for individual features was determined based on assigned molecular formulas as follows [34,35]:

(1) DBE=#C+1−(#H⁄2)+(#N⁄2)

Feature abundances that appear in terms of % Total Abundance throughout this publication were calculated by normalization to the sum of abundances of all filtered features (Abundance > 5000). Data matrices with molecular formulas and DBE assignments can be found in Tables S6 and S7 for neat samples and extracts.

### 2.3. In Vitro Bioactivity Experiments

In total, five organotypic human cell types were used to conduct bioactivity experiments. Organotypic cell types derived from induced pluripotent stem cells (iPSC) were acquired from FUJIFILM-Cellular Dynamics (Madison, WI, USA) and included cardiomyocytes (Cat #R1007, Lot 1299716), endothelial cells (Cat #R1022, Lot 1833921), hepatocytes (Cat #R1027, Lot 7000716), and neurons (Cat #R1013, Lot 1227535). In addition, primary human umbilical vein endothelial cells (HUVEC; Cat #C2519A, Lots 0000433795 and 0000460587, Lonza, Basel, Switzerland). These cell types were selected based on previous studies with petroleum UVCBs that showed them to be most informative for grouping [15,16,17]. All cells were cultured and prepared for treatment based on modified manufacturer protocols (Cellular Dynamics and Lonza) as detailed elsewhere [15,36,37,38,39,40,41,42].

All in vitro experiments were conducted by first preparing a chemical stock plate containing extracts of each substance and all controls (except assay-specific positive controls) in 100% DMSO in a 384-well plate. The compounds in the chemical stock plate were then serially diluted in appropriate cell-specific culture media into working plates at 5× or 2× the desired extract concentration for testing in each cell-specific assay plate. Working plates contained extracts with 2% or 1% DMSO for further dilution to 0.5% or 0.25% (for neurons) DMSO in all assay plates. Thus, in the assay plates, each cell type was exposed to the extracts across five final concentrations: 500 µg/mL, 50 µg/mL, 5 µg/mL, 0.5 µg/mL, and 0.05 µg/mL for neurons (in 0.25% DMSO), or 1000 µg/mL, 100 µg/mL, 10 µg/mL, 1 µg/mL, and 0.1 µg/mL for all other cell types (in 0.5% DMSO). Cell-specific exposure times, controls, phenotypes, and endpoints measured are detailed in Tables S8 and S9. The “method blank” vehicle control [16] was DMSO that was carried through the IP 346 extraction procedure without the inclusion of a petroleum substance.

The experimental design consisted of running a singleton of all the test substance extracts on a single 384-well plate (using only the inner 308 wells) with full concentration response. The inter- and intra-plate controls were included to ensure that the concentration responses observed were not artefacts of the experimental design. Inter-plate controls consisted of running each plate twice; this allowed for a duplicate to be obtained of all substance extracts but also ensured reproducibility between plates. Intra-plate controls were added to ensure that the single values were consistent within a plate. Two olefin substance extracts were selected at random to be present a second time on each plate in a full concentration response representation.

Raw data generated during the in vitro assays was normalized to method blank vehicle control values. The normalized values represent a percent response to the method blank. The normalization was performed for all raw values, including positive/negative controls, using the formula:

(2) $$Normalized\;Value=\left(\frac{Raw\;Value}{Average\;of\;Method\;Blank\;Wells}\right)\times 100$$

To ensure the integrity of the data, several aspects were assessed for each endpoint (data not shown). First, vehicle effects were determined by comparing method blank vehicle, DMSO, and media wells to ensure no effect of the vehicle. The positive cytotoxic control, tetraoctyl ammonium bromide, was also evaluated on all cells. Second, cell type and assay specific positive controls were examined for concentration response with a nonlinear line fit (Hill function) to ensure that the cells were performing as expected from previous publications elsewhere [15,37,38,39,40,41,42]. Third, inter-plate replicate controls were plotted as a scatterplot, with one replicated as the x-value and the other replicated as the y-value. Pearson’s *r* and Spearman’s ρ correlations were calculated, along with *p*-values of significance, and experiments were deemed reproducible if correlations were significant and >0.8. Lastly, intra-plate replicates were plotted as concentration responses with a nonlinear fit (Hill function) to determine if outliers were present.

Upon quality control evaluation, concentration-response data for each endpoint were analyzed to obtain corresponding PODs. Concentration-response data were first normalized to the average of all vehicle treatments (100%). For most of the cell types and phenotypes, a POD was defined as the point where a logistically fitted line departed 10% from the mean of the vehicle control values (EC_10_). Previous investigations have used this POD [39]. Cell- and phenotype-specific PODs are shown in Table S8.

Biological PODs were then analyzed using the Toxicological Prioritization Index (ToxPi) software to generate ToxPi scores [43,44]. First, individual ToxPis were generated for each cell type, with each slice representing a phenotype and equally weighted depending on the number of phenotypes tested per cell type (Table S8). The contribution of each POD element to the ToxPi scores was scaled from lowest bioactivity (ToxPi element = 0) to highest bioactivity (ToxPi element = 1) using the formula:

(3) $$ToxPi\;Value=1-\frac{{log}_{10}(POD)-{log}_{10}({POD}_{min})}{{log}_{10}({POD}_{max})-{log}_{10}({POD}_{min})}$$

Total ToxPi scores for each cell type were then represented in a separate analysis as individual slices to generate an overall ToxPi depicting all cell types. All substances were included as “available chemicals” in the software settings, and each cell type tested was displayed as an individual pie slice. The distribution for each slice was log-scaled and equally weighted in its contribution to the overall ToxPi.

### 2.4. Clustering of Substances Using IMS-MS and Bioactivity Data

Grouping of LBN and RO categories as well as human health subcategories for both biological and chemical data was conducted using unsupervised hierarchical clustering via *hclustfunc* in *heatmaply* and *gplots* packages in *RStudio*.

### 2.5. Predicting Bioactivity Based on IMS-MS Chemical Profiles

For prediction of the bioactivity from the individual chemical features in neat or extracted samples, an extension of the penalized ridge regression approach as developed in [45] was used. Briefly, the approach performs multivariate ridge regression for the multivariate linear model *Y* = *XB* + error, where *Y* (*n*X*m*) and *X* (*n*X*p*) are scaled bioactivity and feature matrices with dimensions shown, and *B* (*p*X*n*) is a coefficient matrix. Here, *n* is the sample size of substances, *m* is the number of bioactivity measurements, and *p* is the number of features used in the predictions. Briefly, one can envision the bioactivity data as a multi-dimensional readout *Y* with *n* rows, where each row included data for one endpoint, cell-specific overall ToxPi scores, or an overall ToxPi score incorporating all cell types together. The matrix had 25 columns for each chemical, classified by their category. A chemical predictor matrix for neat substances *X* had 225 rows (features comprising >1% of at least one sample) and 25 columns (one per sample). Similarly, a separate chemical predictor matrix for DMSO extracts had 212 rows (features > 1%) and 25 columns. Prior to fitting, all data columns were centered and scaled to unit variance for comparability and to ensure no predictor dominated simply due to scale differences.

The fitted model is truly multivariate because a single tuning penalty *λ* is applied, with $\hat{B}={\left(X^{T}X+\lambda I\right)}^{-1}(X^{T}Y)$ (which is the ridge regression approach) and final prediction $\hat{Y}=X\hat{B}$. *λ* was evaluated on a grid such that log_10_(*λ*) varied uniformly from −1.0 to 6.0 in increments of 0.1. Evaluations were performed using leave-one-out cross validation, i.e., prediction for elements of *Y* from the *i*^th^ sample used coefficients obtained after removing the *i*^th^ sample, to avoid overfitting. The selection of the tuning parameter was performed to give the minimum mean squared prediction error. Final predictions were returned to the original *Y* scale by multiplying each column by the original standard deviation and adding the original mean. The entire procedure was then run again to predict features by reversing the assignment of *X* and *Y* matrices.

As a measure of model fit for each bioactivity feature, the Pearson correlation *r* between the observed bioactivity values and the values predicted in cross-validation was used. Standard cross-validation principles [46] rely on the fact that the test sample (which is singular under leave-one-out cross-validation) is held out for model training, and thus each test set prediction is often treated as independent of the training set. However, a subtle internal dependency can arise due to the scaling of *X* and *Y*, which is performed once. In addition, our final prediction tuning parameter was selected once, outside of the cross-validation loop. Thus, as a conservative measure without requiring complicated double cross-validation loops, *p*-values for the predicted-observed *r* using a permutation procedure were computed. A total of 1000 permutations of the sample indices in *Y* and *X* were performed, with the mean and standard deviations of the (null) *r* values used to compute a statistic *z* = (*r* − E(*r*))/SD(*r*), which was compared to a standard normal distribution in a two-sided test. The resulting *p*-values for each bioactivity feature were then corrected for multiple comparisons by computing the Benjamini–Hochberg *q*-value [47] using the *R* v4.1 *p.adjust* package.

## 3. Results and Discussion

**Figure 1:**
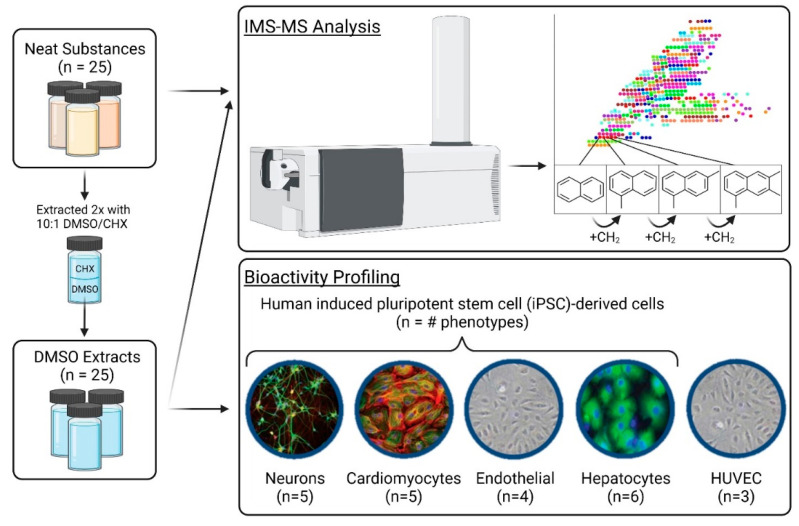
Schematic of the overall experimental design depicting the chemical analytical and bioactivity profiling of neat substances and their respective DMSO extracts (*n* = 25).

The overall experimental workflow is shown in Figure 1. Both neat substances (two manufacturing categories, 25 substances in total, Table 1) and their respective DMSO extracts were analyzed using nontarget IMS-MS. DMSO extracts of each test substance were used for in vitro assays across four induced pluripotent stem cell-derived cell types (cardiomyocytes, endothelial cells, hepatocytes, and neurons) and human umbilical vein endothelial cells (HUVEC).

### 3.1. Compositional Characterization and Similarity between Test Substances

**Figure 2:**
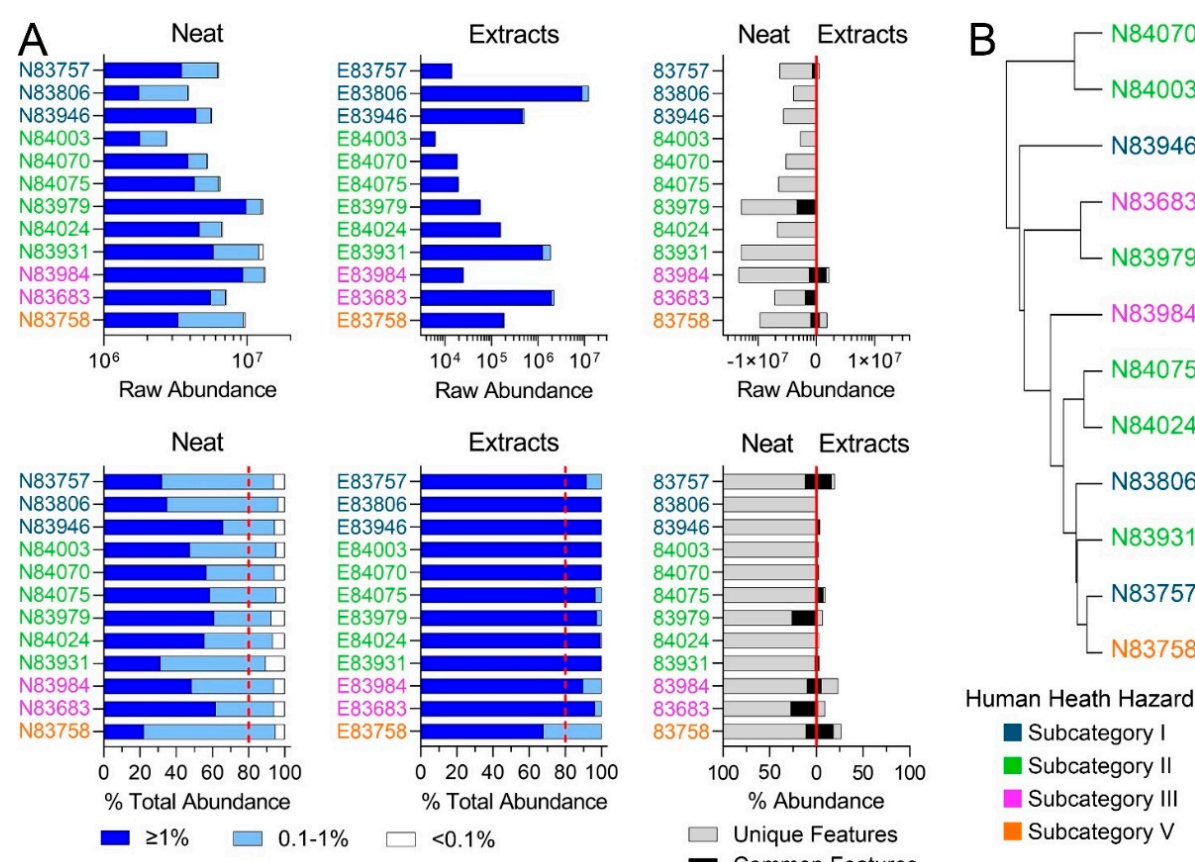
(**A**) Feature abundances for Low Benzene Naphthas category. The top row depicts the raw abundances of features detected for neat products, product extracts, and the abundance of features characterized by the same molecular formula in neat and corresponding extract substances. The bottom row depicts the same features normalized to the total abundance of features per substance. Dark blue bars denote features present at >1%, light blue bars denote features present at 0.1–1%, and white bars denote features present at <0.1% abundance. Dotted red lines refer to ECHA’s 80% minimum threshold [14] for UVCB characterization. The third plot in each row shows features present in both the neat samples and DMSO extracts (black bars) and features unique to each (grey bars). (**B**) Hierarchical clustering portraying the chemical similarity of LBN neat substances based on IMS-MS profiles. Substances closer together have the most similar chemical profiles. Colors indicate pre-assigned health hazard groups.

**Figure 3:**
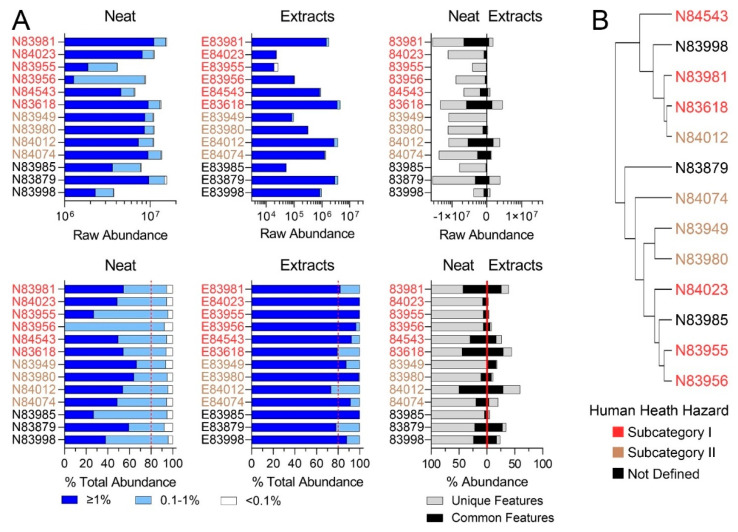
(**A**) Feature abundances for the Resin Oils category. The top row depicts the raw abundances of features detected for neat products, product extracts, and the abundance of features characterized by the same molecular formula in neat and corresponding extract substances. The bottom row depicts the same features normalized to the total abundance of features per substance. Dark blue bars denote features present at >1%, light blue bars denote features present at 0.1–1%, and white bars denote features present at <0.1% abundance. Dotted red lines refer to ECHA’s 80% minimum threshold [14] for UVCB characterization. The third plot in each row shows features present in both the neat samples and DMSO extracts (black bars) and features unique to each (grey bars). (**B**) Hierarchical clustering portraying the chemical similarity of RO neat substances based on IMS-MS profiles. Substances closer together have the most similar chemical profiles. Colors indicate pre-assigned health hazard groups.

Regulatory guidelines require compositional characterization and assessment of the variability between substances to (1) determine the applicability domain of a category, (2) confirm membership in that category, and (3) establish a basis for read-across of toxicological properties [14]. To fulfil these criteria for the substances tested herein, the chemical profiles obtained with IMS-MS nontarget analysis were first analyzed separately within the LBN and RO categories (Figure 2 and Figure 3). Figure 2A shows the profiles of the substances originally identified as belonging to the LBN category, both in terms of the raw abundance of various constituents and as a percentage of the total abundance within each sample. A complete list of sponsored streams is available in the US EPA Screening-Level Hazard Characterization for LBN (see access links to the documents in Table S10) [26]. According to US EPA [26], the LBN category comprises 12 unique chemical identifiers and 9 production streams; in this study, substances were available for experiments that represented 10 identifiers and 8 production streams.

First, raw abundance profiles showed the complexity of the substances and the variability in their composition within and across human health subcategories (Table 1). The substances belonging to subcategory I, high toluene streams, were the least complex of the LBN substances tested in terms of the overall raw abundance of the constituents. This was expected, because substances belonging to this subcategory should be composed of C7–C8 range constituents, while the LBN category as a whole consists *“primarily of C7 to C12 aromatic and cycloaliphatic hydrocarbons”* [26]. Similar observations were made when the data were expressed in percent abundance, although abundance normalization demonstrates a more homogeneous LBN category than raw abundances (Figure 2A, bottom). The composition of DMSO extracts had little overlap with the corresponding neat products, both in terms of raw and normalized abundance. Figure 2B shows hierarchical clustering of the samples using analytical data. It is evident that while some substances from the same human health subcategory cluster together, others are not sufficiently similar using the chemical compositional profiles from IMS-MS analyses.

Similar observations were made for RO substances (Figure 3A). A complete list of sponsored streams is available in the US EPA Screening-Level Hazard Characterization for RO (see access links to the documents in Table S10) [27]. The US EPA specified that this category includes 11 unique chemical identifiers in 9 production streams; herein, we tested substances from 6 identifiers and 5 production streams. Three RO substances that were available for this study could not be defined into one of the existing subcategories. Raw abundance profiles again demonstrated the variation in chemical composition among substances. Subcategory I exhibited the most variation, while subcategory II exhibited the most similar substance profiles. This was supported by hierarchical clustering (Figure 3B). Corresponding DMSO extracts showed the variation between substances and, although to a greater extent than for LBN samples, still captured a very small fraction of the corresponding neat substances (Figure 3A).

Second, the most recent regulatory guidance on substance chemical characterization [14] details the extent of information needed for UVCBs, including constituent identities and concentrations. Compounds present at ≥1% abundance must comprise at least 80% of the sample to warrant more extensive characterization of molecular structures for hazard evaluation. For cases where the 80% threshold is not met, it is *“not technically possible or impractical”* to identify the individual constituents, and *“structural similarity must be demonstrated by other means.”* “Other means” may include pre-existing information on starting materials and manufacturing processes or fingerprinting analysis; however, analytical methods must enable *“the provision of information on a sufficient proportion of constituents… [to cover] >95% of the constituents of a substance”* [14]. Thus, for analyses in Figure 2 and Figure 3, constituents were classified as comprising ≥1%, 0.1–1%, and <0.1% of a sample for all LBN and RO neat products and extracts. For both categories, features of ≥1% abundance in the neat substances did not meet the ECHA’s 80% threshold, meaning that the use of “other means” to characterize the composition of the neat substances may be justified. However, for toxicity testing, it is equally important to characterize the DMSO extracts used to expose the substances. Features of ≥1% abundance in the extracts constituted >80% of each substance, meaning that constituents of concern at concentrations below 0.1% may also need to be identified using additional analytical techniques. Without analytical reference standards to confirm the structural identities of these low-concentration constituents, analyses herein were restricted to putative molecular formulae. Ultra-high-resolution techniques or structure-based modeling approaches may be better suited to confirm the identities of these constituents. Still, the number of species present in each sample <0.1% is vast, and structural identification of all constituents of concern would be a daunting task.

**Figure 4:**
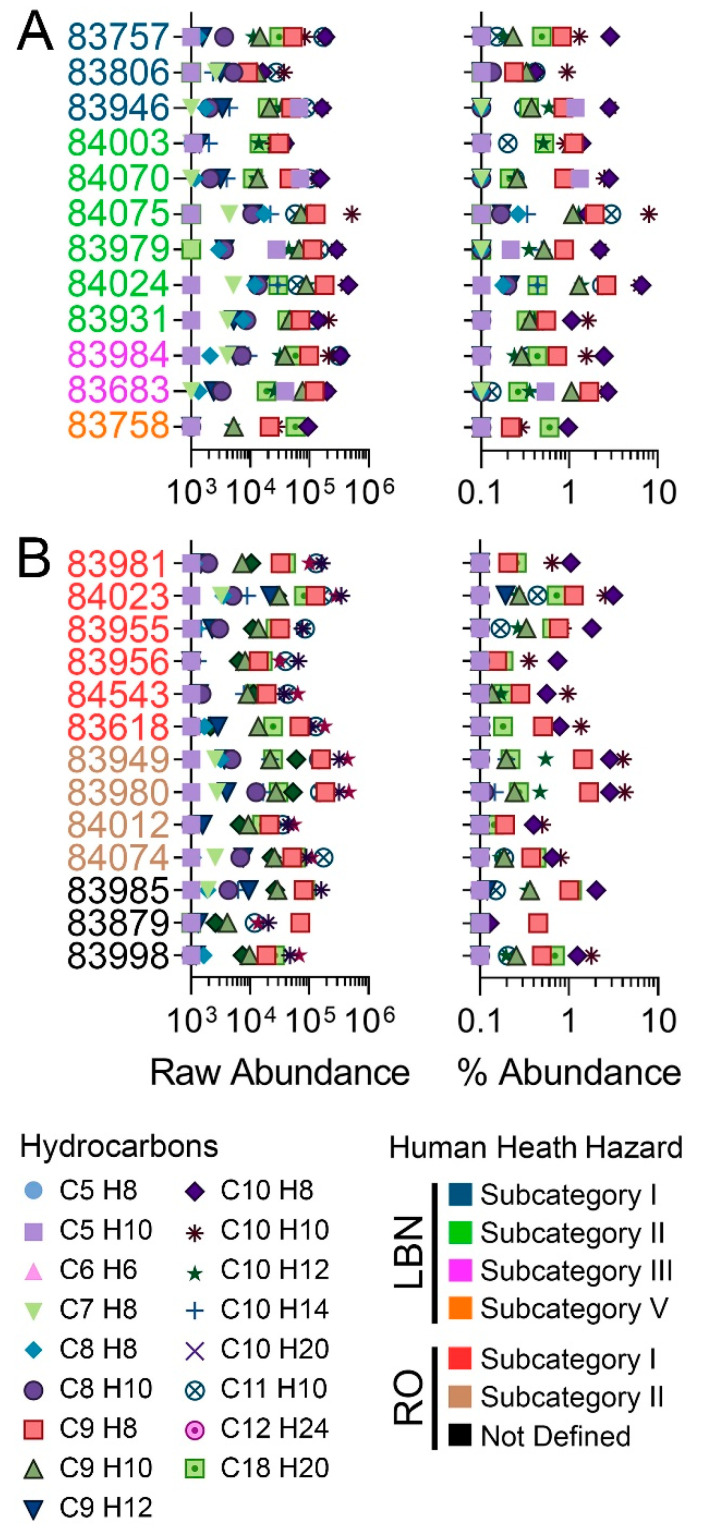
Total feature raw (left) and percent (right) abundance of unique molecular formulas representing typical constituents for LBN (**A**) and RO (**B**) categories, see color legend. Constituents were selected based on the substance profiles [48,49]. More detailed analysis can be found in Tables S11 and S12.

Third, the composition of the constituents obtained using nontarget IMS-MS was compared to the typical constituents reported in REACH Category Identity Profiles [48,49], information that is derived using traditional analytical methods (Table 2 and Table 3). These data are typically reported for a limited number of the most abundant constituents, which are known to vary among registered substances. The reported LBN constituent list [48] includes 45 compounds with CAS numbers mapping to 18 unique molecular formulas (Table 2). Figure 4 shows the raw and relative abundances for IMS-MS-observed constituents that matched these formulas. As expected, their abundance varied among substances within each category. We compared the reported typical ranges with those from IMS-MS analyses (Table 2). Even though the data was obtained using different analytical methods and on different samples, we reason that by normalizing abundances as a percent of the total sample, it is possible to perform meaningful comparisons. Overall, IMS-MS data were well within the typical range for all constituents, with the maximum observed concentration for any single constituent being toluene at 6.7%. Still, because the IMS-MS approach provides higher resolution and more individual constituents are detected, the relative abundances were lower than those typically reported using other techniques. Seven molecular formulae spanning 10 CAS numbers were below the limit of detection.

Based on IMS-MS data, LBN substances exhibited similar relative abundances of the reported constituents, and little variation was observed between human health subcategories [26]. Subcategory I substances are typically distinguished by high toluene content, although other, higher *m*/*z* compounds at a higher abundance than toluene (C7H8) for substances 83757, 83806, and 83946 were detected in this study. Subcategory II substances are expected to contain toluene, ethylbenzene (C8H10), and xylenes (C8H10, all isomers included); these were all detected by IMS-MS in relatively high amounts (though not as high as C9-C10 compounds), although ethylbenzene and xylene isomers could not be distinguished without analytical reference standards. Typical components for subcategory III include toluene, xylene isomers, styrene (C8H8), and naphthalene (C10H8). Naphthalene was the constituent detected by IMS-MS in the highest abundance for both samples belonging to subcategory III (2.4–2.7%), while the other component chemicals were detected at a lesser abundance (~0.05%). Subcategory V has no reported specific constituents apart from being described as *“C9+ from o-xylene unit”*; sample 83758 fit this description, and C9H8, C9H10, C10H8, C10H10, C10H12, and C18H20 were all listed constituents of highest abundance [26].

The reported RO constituent list [49] included constituents with 38 CAS numbers that mapped to 20 unique molecular formulae (Table 3); these are expected to comprise between 0% and 80% of any RO substance. Constituents matching twelve of these unique molecular formulae were detected by IMS-MS in RO substances tested herein, ranging in abundance from 0% to 3.14% (naphthalene; Figure 4, Table 3). Eight molecular formulae representing 11 CAS numbers were not detected by IMS-MS. Unlike human health subcategories for LBN, RO subcategories are distinguished mostly by varying levels of dicyclopentadiene (DCPD, C10H12). As expected, DCPD was one of the highest detected constituents using IMS-MS across RO substances, both in subcategories I (high DCPD) and II (low DCPD). Samples for substances representing subcategory III, for which methylcyclopentadiene dimer (MCPD; C12H16) is an additional supporting chemical [27], were not available for this study. More detailed analyses for Figure 4 can be found in Tables S11 and S12 for LBN and RO, respectively.

### 3.2. Bioactivity Profiling

**Figure 5:**
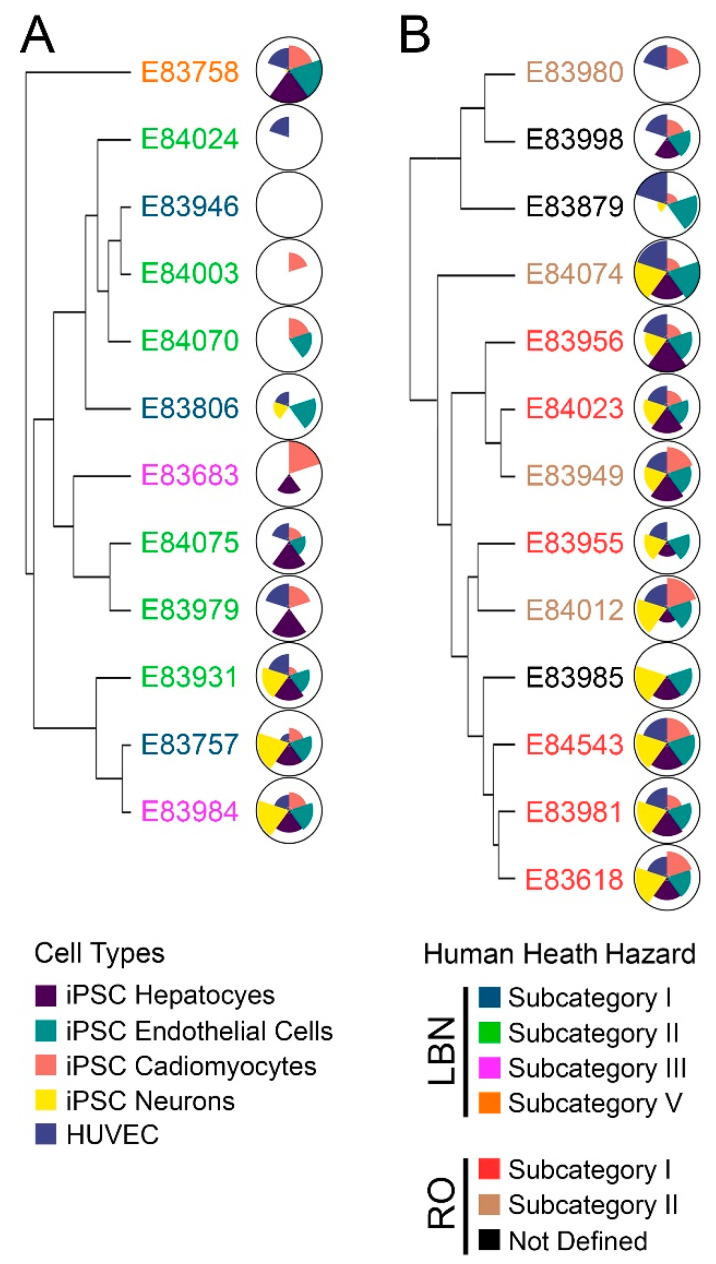
(**A**) Hierarchical clustering based on bioactivity profiles for LBN DMSO extracts. The name of each substance is colored by the prescribed health hazard group. Corresponding ToxPi diagrams depict overall substance toxicity; each slice represents one cell type, including all assessed phenotypes. Cell types tested include iPSC-derived hepatocytes (purple), endothelial cells (green), cardiomyocytes (pink), neurons (yellow), and HUVEC (blue). Larger pie slices indicate greater toxicity for that substance and cell type. (**B**) Hierarchical clustering based on bioactivity profiles for RO DMSO extracts. Substance names are again colored by prescribed health hazard groups, and respective ToxPi charts show an overall greater toxicity of RO substances as compared to LBN substances.

Characterizing the composition of UVCBs is critical to establishing structural similarity and the applicability domain of a category [14]. Still, the inherent variability between substances presents uncertainty that may be addressed through the evaluation of the bioactivity of the individual substances. Herein, bioactivity profiling, i.e., testing of the concentration-response effects of the DMSO extracts of the petroleum UVCBs on various human cells and endpoints, was conducted. This analysis aimed to determine whether (i) similarity in bioactivity would be observed within each category and (ii) similar bioactivity profiles would be concordant with chemical similarity from IMS-MS data. The ToxPi approach for integrating bioactivity data across different phenotypes and cell types [15,16,50] is a common method for visualization and ranking of substances. Here, the data from 20 phenotypes across 5 cell types (Table S8) were integrated by constructing substance-specific ToxPi to represent bioactivity, where one pie slice equates to the overall ToxPi score derived for each cell type. ToxPi profiles were assembled within each tested category, LBN and RO, whereby the bioactivity is relative within that category. Greater bioactivity (i.e., lower POD) is represented by a larger ToxPi score and a bigger pie slice. Unsupervised hierarchical clustering was then used to assess the similarity between the bioactivity profiles of different substances within each category (Figure 5). Overall, RO substances (Figure 5B) exhibited greater bioactivity than LBN substances (Figure 5A). This finding corroborates previous reports, which showed greater bioactivity of higher carbon-range vacuum and hydrotreated gas oils as compared to lower carbon-range straight-run gas oils [15]. Similar to the observations with chemical composition (Figure 2 and Figure 3), there was some, albeit not complete, similarity in bioactivity profiles within each human health subcategory.

In the LBN category, subcategory II exhibited the greatest bioactivity similarity. Some of the substances had lower bioactivity (E84024, E84003, and E84070) as compared to others (E84075, E83979, and E83931). This result is concordant with the data on chemical composition; samples 84003 and 84070 are two of the least complex LBN substances tested, and they also exhibited few effects in vitro. Similarly, samples 84075 and 83979 were of comparable chemical complexity and elicited similar bioactivity profiles. iPSC-derived neurons and hepatocytes were the most affected cell types across the LBN substances.

In the RO category, subcategory I exhibited the most similarity in bioactivity profiles; five out of the six samples assigned to subcategory I demonstrated bioactivity in all cell types tested. The sixth sample (E83955) was bioactive in four out of five cell types, albeit to a lesser extent. These results were also generally concordant with the chemical composition data in Figure 2 and Figure 3; subcategory I substances 83956 and 84023 were closely related, while 84543 and 83981 also exhibited compositional concordance. Bioactivity was observed more consistently across all cell types for RO substances; still, iPSC-derived endothelial cells, neurons, and hepatocytes were the cell types for which bioactivity was observed most often.

### 3.3. Comparison of Bioactivity and Chemical Composition

**Figure 6:**
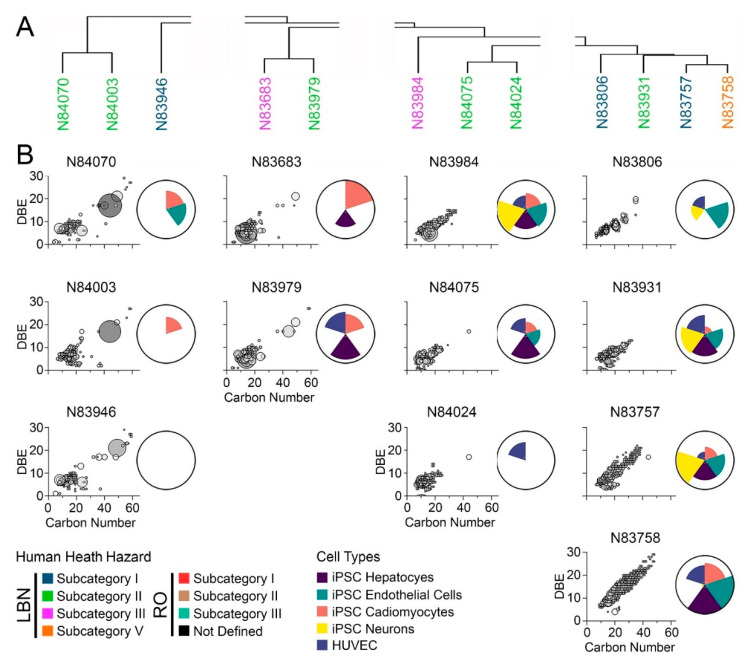
(**A**) Hierarchical clustering based on chemical profiles of neat LBN substances. Substance names are colored by prescribed health hazard groups. (**B**) IMS-MS chemical profiles depicted as carbon number versus double bond equivalence. Larger bubble sizes and a darker grey color depict more abundant features. Adjacent ToxPi charts show overall bioactivity across iPSC-derived hepatocytes (purple), endothelial cells (green), cardiomyocytes (pink), and neurons (yellow), as well as HUVEC (blue).

**Figure 7:**
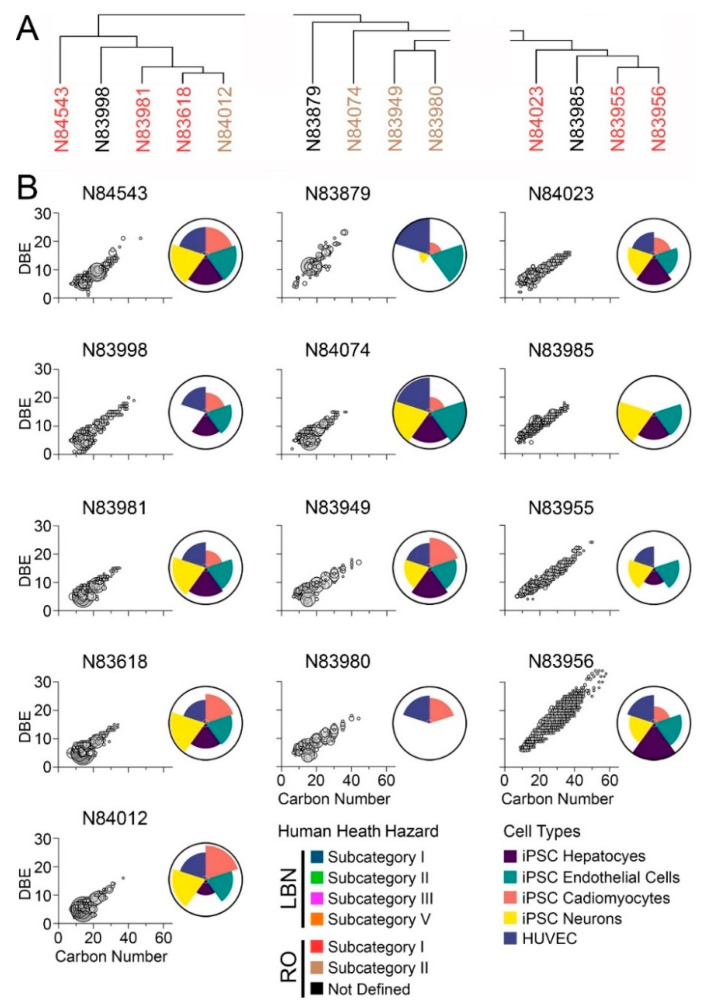
(**A**) Hierarchical clustering based on chemical profiles of neat RO substances. Substance names are colored by prescribed health hazard groups. (**B**) IMS-MS chemical profiles depicted as carbon number versus double bond equivalence. Larger bubble sizes and a darker grey color depict more abundant features. Adjacent ToxPi charts show overall bioactivity across iPSC-derived hepatocytes (purple), endothelial cells (green), cardiomyocytes (pink), and neurons (yellow), as well as HUVEC (blue).

Human health evaluations for petroleum UVCBs are typically based on substance grouping using physio-chemical properties and manufacturing processes, followed by an assessment of possible hazards by several constituents. The bioactivity profiling described above (Figure 5) grouped substances based on similarity in bioactivity, but the grouping was not fully concordant with existing HPV categories; therefore, “substance similarity” was examined using both chemical profiles and bioactivity. Specifically, the objective of this study was to assess chemical and in vitro data together to determine whether chemical composition may align with trends in bioactivity (Figure 6 and Figure 7). First, the overall chemical composition clustering of samples in the LBN category (Figure 2) was split into four sub-groups based on clustering (Figure 6A). To visualize the hydrocarbon composition of each substance, the carbon number range was plotted versus double bond equivalence (DBE) and abundance (Figure 6B). This typical data presentation for petroleum UVCBs allows a visual assessment of the complexity of each sample, as well as the range of hydrocarbon types that are present. Aromaticity, measured by DBE, varied from a minimum of 1 (low aromaticity, likely olefin or alkane species) to a maximum of 30+ (highly aromatic species). Overall, the chemical profiles of most LBN samples were within the expected C7–C12 range; however, many samples contained an appreciable number of constituents in the C40 range that are aromatic. Generally, samples with a higher carbon number range exhibited greater bioactivity across all cell types tested. The first subgroup (N84070, N84003, and N83946) included the least bioactive substances of all tested samples; based on their compositional signatures, these samples were clustered based on the high abundance of C40+ constituents. The second subgroup (N83683 and N83979) exhibited a high abundance of <C20 constituents, as well as some that were >C40 (although these were not as highly abundant as in the first subgroup). Between the two substances in this subgroup, the most bioactivity was contributed by iPSC-derived cardiomyocytes and hepatocytes. The third subgroup (N83984, N84075, and N84024) displayed the most chemical similarity between N84075 and N84024, although these substances only had hepatocyte bioactivity in common. N83984 had a chemical abundance distributed over a wider carbon range (up to C40) and exhibited greater bioactivity in all cell types. Finally, the last subgroup (N83806, N83931, N83757, and N83758) presented the greatest chemical variability and carbon number range when compared to the other LBN samples. The three substances (N83931, N83757, and N83758) with the largest carbon number range and high levels of aromatic species exhibited some of the highest bioactivity, which was especially notable in iPSC-derived neurons, endothelial cells, and hepatocytes.

The same analyses were conducted for RO substances (Figure 7). Three subcategories were examined (Figure 7A). Most of the samples had the greatest number of constituents (Figure 7B) in the C7–C20 range; however, all substances had constituents in the C20–C40 range, and one substance (N83956) extended to C50+. Like LBN substances, greater bioactivity across all cell types tested was generally associated with a larger carbon number range. All RO substances exhibited a larger carbon number range than LBN substances (except for N83757 and N83758). Substances in the first RO subgroup (N84543, N83998, N83981, N83618, and N84012) exhibited bioactivity in all cell types tested except sample N83998, which was not bioactive in iPSC-derived neurons. Three of these substances belong to human health subcategory I (N84543, N83981, and N83618). Of the four substances belonging to the second subgroup (N83879, N84074, N83949, and N83980), three were in human health subcategory II and had generally comparable chemical profiles; still, N83980 exhibited bioactivity only in iPSC-derived cardiomyocytes and HUVEC, whereas N84074 and N83949 exhibited bioactivity in all cell types tested. Similar conclusions could be drawn for the third subgroup (N84023, N83985, N83955, and N83956); these substances are members of RO human health subcategory I and showed considerable overlap in chemical composition. Despite the difference in carbon number ranges between N84023 and N83956, their bioactivity profiles shared a closer resemblance to each other.

### 3.4. Determining What Chemical Constituents May Be Associated with Bioactivity

Data on PAC content with 3+ aromatic rings is conventional analytical chemistry-based information that is used to judge the potential health hazards of petroleum UVCBs; higher PAC content is assumed to have higher bioactivity [51,52]. However, regulatory bodies such as ECHA are typically hesitant to rely on these data alone in hazard evaluation, reasoning that PAC content may not necessarily represent the entire bioactive fraction [10]. It was also argued by ECHA that such a broad characterization does not provide enough information to justify the application of read-across [10]. Indeed, considerable heterogeneity in both chemical composition [25] and bioactivity [15,16,17] of substances within current petroleum UVCB categories, based on the physio-chemical properties and manufacturing process, has been previously observed; therefore, the findings presented in Figure 2, Figure 3, Figure 4, Figure 5, Figure 6 and Figure 7 for LBN and RO categories are not unexpected. While such heterogeneity in both overall chemical composition and bioactivity cannot be used directly to justify similarity between substances in each category, determination of whether there may be statistically significant associations among specific chemical constituents and bioactivity phenotypes has not been previously attempted for petroleum UVCBs.

**Figure 8:**
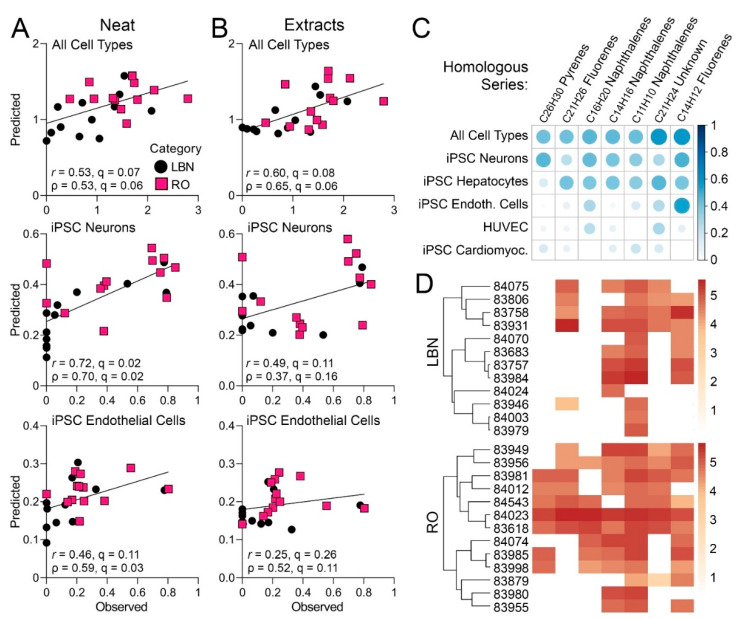
(**A**,**B**) Each scatterplot shows bioactivity (*y*-axis) for the overall ToxPi score (top), iPSC-derived endothelial cells (middle), and iPSC-derived neurons (bottom) as predicted from the chemical profiles of neat (**A**) and corresponding extracts (**B**). Observed bioactivity is shown on the *x*-axis. Bioactivity prediction was conducted using the penalized regression approach described in Methods. The predicted values were obtained by leave-one-out cross validation, where the prediction model was developed with each sample left out of analysis, and the model applied to the features of the held-out sample. The most informative validations were chosen with the highest prediction r (Pearson coefficient) and the lowest q (false discovery rate value). (**C**) Correlation plot depicting the hydrocarbon compounds from neat samples that were most significantly predictive of the overall ToxPi score based on cross-validation analyses. Bubble size represents the Pearson correlation between feature abundance and ToxPi score overall as well as for individual cell types. Positive correlations are shown in blue, whereas negative correlations are shown in red. (**D**) Heatmap depicting the relative abundance of each feature in each sample tested. A darker color indicates higher abundance.

Therefore, machine learning was used to predict overall and cell type-specific bioactivity from the IMS-MS chemical profiles for the tested substances (Figure 8). This approach has previously been used to provide a refined analysis of bioactive components in case studies of other complex substances [45] and mixtures [53]. Even though neither chemical composition, nor bioactivity data separately replicated existing categories/sub-categories of the tested substances, the overall bioactivity of each sample was found to be strongly associated (multiple testing-corrected *q-*value <0.1) with the chemical profiles of both neat and DMSO-extracted samples (Figure 8A and B, top). Interestingly, the data from iPSC-derived neurons and endothelial cells was also strongly associated with the chemical profiles of the neat substances (Figure 8A, middle and bottom), but not of the DMSO extracts (Figure 8B, middle and bottom). Next, it was determined what constituents in the neat samples were most influential in this multivariate prediction analysis (Figure 8C). Of the seven constituents that were significantly associated with bioactivity, all were high-molecular-weight PAC belonging to homologous series with pyrene, fluorene, or naphthalene. Only one constituent could not be identified with high confidence using a workflow for IMS-MS data analysis of petroleum substances [18]. Table S13 shows a list of potential names that could be assigned to the seven hydrocarbon features driving bioactivity and their corresponding hazard classifications. Further, the relative abundance of these constituents in each tested sample (Figure 8D) was compared. It was found that there was an overall higher abundance of these constituents in RO substances as compared to LBN substances, supporting the previous observation that RO substances were generally more bioactive.

## 4. Discussion

This study is novel because it used new analytical and toxicological approaches to examine both the chemical composition and biological effects of complex petroleum UVCBs. Samples were from two HPV categories, and this study aimed to determine the extent of chemical and bioactivity similarity among substances that have been previously assigned to these categories using physio-chemical properties and manufacturing process information. The main questions of this study were four-fold: (1) To what extent can petroleum UVCBs be characterized using novel analytical methods such as IMS-MS to meet the most recent ECHA advice on substance characterization for read-across [14]? (2) How much *chemical* variability is to be expected within and between existing LBN and RO manufacturing categories? (3) How much *biological* variability is to be expected within and between existing LBN and RO manufacturing categories? Additionally, (4) What constituents are potential drivers of bioactivity in complex petroleum UVCBs?

First, it was found that in the DMSO extracts (but not in the neat substances) in the RO and LBN manufacturing categories, the sum of constituents present in amounts ≥1% of the overall substance was above the 80% ECHA threshold [14]. This means that additional analyses need to be performed to further identify the constituents of concern below 1%; for this, higher resolution analytical instruments such as Orbitrap and Fourier transform ion cyclotron resonance (FT-ICR) mass spectrometry (MS) may be more suitable [5,19]. In addition, subsequent application of targeted chromatographic approaches would also be needed to confirm the structural identities of identified constituents of interest [5,54,55,56].

Second, broad chemical concordance was observed for substances belonging to the same category; however, considerable variability was observed between substances in the same category and even subcategory. This was likely a result of inherent substance variability or reaction byproduct impurities from manufacturing processes. While compositional variability is to be expected, recent advice from ECHA calls for the characterization of such variability. Not only is there a need to provide compositional characterization of the substances identified by different CAS RN but grouped into a category, but also characterization of the variability of the same product across manufacturing batches and refineries [14]. The analysis of at least five independent (i.e., production batch) samples from all registrants of a substance is the most recent threshold proposed by ECHA [14]. To establish this, novel analytical techniques such as IMS-MS, Orbitrap-MS, and FT-ICR-MS are most appropriate [5]. A recent study showed that detailed chemical compositional data on petroleum UVCBs obtained from IMS-MS can provide the information necessary for hazard and risk characterization in terms of quantifying the variability of the products in a manufacturing category, as well as in subsequent production cycles of the same product [25].

Third, similarity in bioactivity was observed within the overall LBN and RO categories; however, less concordance was evident within previously proposed HPV human health subcategories. This observation is similar to that from a larger study of other petroleum UVCBs, where 141 substances spanning 6 product categories were tested in 15 human organotypic cell types to investigate substance similarity using both bioactivity signatures [16] and transcriptomic profiles [17]. These studies showed that the bioactivity and transcriptomic data correlate strongly with the PAC content of each substance and can be used to rank overall categories in a way similar to that using other hazard data (typically from animal and genotoxicity studies); however, they cannot be used to substantiate existing groupings. These data are still highly informative, as a combination of bioactivity and transcriptomic data could be integrated to make decisions as to the selection of class-representative worst-case petroleum UVCBs for subsequent evaluation in vivo [57].

Fourth, this study is also informative in terms of the hazard evaluation of petroleum UVCBs. Due to the chemical complexity of petroleum UVCBs, there is no harmonized methodology for their risk assessment; both whole mixture and constituent-based approaches can be used [58,59,60]. The constituent-based approach is most commonly used for petroleum UVCBs [61,62]; however, the approaches to the selection of the chemical constituents of interest are yet to be standardized [4,63,64]. Furthermore, petroleum UVCBs are typically tested as the whole substance (in vivo) or as a DMSO extract (in vitro), rather than as individual constituents or groups of constituents [65]. The results presented herein are consistent with the historical observations that the potential hazards of petroleum UVCBs are largely determined by their PAC 3–7 ring content [66,67,68] and previous observations that PAC content is the strongest “driver” of in vitro bioactivity [16,17,69]. In addition, this study also provides specific details on what constituents, rather than PAC 3–7 overall, are most strongly associated with in vitro bioactivity. Such an approach, assessing relationships between high-dimensional chemical profiles and multi-dimensional bioactivity phenotypes, is informative for defining constituents of interest for component-based risk assessment of petroleum UVCBs. This is especially beneficial in scenarios such as environmental disasters, where exposure assessment and hazard evaluation are time sensitive [50,53].

This study is not without limitations. The availability of samples, a common challenge in studies of large-volume produced substances, limited our ability to characterize the intra-category and sub-category variability. Even though we tested 25 samples that were representative of two manufacturing categories and multiple sub-categories within them, the desired replication was lacking. Prior studies showed that a single sample per category may not provide adequate information to capture the individual category characteristics [70]. Updated ECHA advice also addressed this limitation, specifying that constituent concentrations in *“at least five independent samples of the substance…from different production batches…as produced by all the registrants”* must be included to characterize the variability [14]. However, obtaining samples for the analysis of petroleum UVCBs is a well-known challenge that cannot be easily addressed because samples need to be provided by the individual manufacturers and cannot be commercially procured from standard chemical suppliers. Some studies have begun to address compositional variability within production batches [25]; still, additional investigation is warranted to examine variability in bioactivity within production batches as well.

Our study used one analytical approach to characterize the chemical composition of tested substances; however, products of petroleum refining are highly complex, and both separation, ionization, and detection methods may affect the molecules that are identifiable using each technique [5,19,71]. Therefore, the analytical results presented herein should be interpreted with caution. For example, we reason that while they may be used for the purpose of relative comparisons among substances and categories, they should not be used to infer the exact chemical composition or absolute concentrations of the individual constituents.

In addition, DMSO extraction, a widely used method to enable testing complex petroleum substances [51,72], captures only a fraction of the neat substance. This is a concern for regulators, who maintain that solvent extraction may restrict the bioactive fraction to only constituents that are soluble in biocompatible solvents such as DMSO [10]. Recent developments in the field have therefore adapted alternative dosing techniques as potential solutions to enable more high-throughput in vitro testing [73,74,75,76,77,78], and future studies of petroleum UVCBs may utilize these alternative approaches for delivering the substances in small-volume in vitro methods.

Another well-recognized challenge of using in vitro bioactivity for hazard-based evaluations of chemicals is the translation of in vitro results to apical in vivo phenotypes. The complex composition of UVCBs makes it difficult to conduct traditional in vitro-to-in vivo extrapolation from bioactive concentrations to human exposures [79]. It is still debated as to whether bioactivity should be used only for screening and prioritization [80], for grouping and read-across [81], or to establish health-protective points of departure for screening-level assessments [82]. The use of in vitro bioactivity data in regulatory decision-making is rapidly evolving, and regulators currently indicate that the results of cell-based studies should be confirmed with additional assays, including studies in animals [83].

## 5. Conclusions

Overall, this study demonstrates the benefits of simultaneous assessment of both chemical composition and bioactivity when evaluating the potential hazard properties of petroleum UVCBs. We found that based on the samples analyzed herein, existing categories, based largely on the manufacturing considerations and intended future uses of these products, may be considered heterogeneous in terms of their composition and bioactivity. While additional work is needed to evaluate a larger compendium of substances, including different manufacturing batches of the same substance and testing alternative in vitro delivery methods for these “difficult to test” substances, we conclude that an approach that combines chemical composition and bioactivity data is sensible. These complementary data streams provide information that will enable a more comprehensive and confident characterization of similarities, differences, and variability between and within manufacturing categories of petroleum UVCBs.

## Figures and Tables

**Table 1:** Petroleum UVCBs from lower olefin categories that were tested in this study.

Sample ID *	Sponsored Stream *	CAS RN ^#^	Human Health Hazard Subcategory ^#^
**Low Benzene Naphthas**			
83757 83806	Pyrolysis C7	68527-23-1 68478-10-1	Group I: High Toluene Streams
83946	Pyrolysis C7-C8	68527-23-1 68919-15-3	
84070 84003	Hydrotreated C7-C8		Group II: Mixed Aromatics Streams
84075	Hydrotreated C7+	64742-48-9	
84068	Hydrotreated C8-C10	68512-78-7 64742-48-9	
83979 84024	Hydrotreated C7-C12	64742-48-9 68516-20-1	
83931	Solvent Naphtha	68512-78-7	
83984 83683	Pyrolysis C7-C12	68516-20-1 64742-83-2 68746-45-9	Group III: Pyrolysis C7-C12
83758	C9+ from o-xylene	68333-88-0 68553-14-0	Group V: C9+ from o-xylene unit
84082	Solvent Naphtha	68512-78-7	Not Defined Properly
**Resin Oils and Cyclodiene Dimer Concentrates**			
83981	DCPD, High Purity	77-73-6	Group I: DCPD High Purity & Related Streams
84023	High DCPD Resin Oil	68477-54-3 68477-40-7	
83955	Distillates (petroleum), steam cracked. C8-C12; High DCPD Resin Oil	68477-54-3	
83956	Resins Distillates (petroleum), cracked stripped steam cracked. C10-C12	68477-40-7	
84543	HYDROCARBONS, C5-RICH, DICYCLOPENTADINE Resin; DCPD Concentrate Distillates (Petroleum) steam cracked C5-C12 fraction	68527-24-2	
83949	Low DCPD Resin Oil	68477-54-3 68516-20-1	Group II: Low DCPD Resin Oil & Resin Former
83980 84012 84074	Low DCPD Resin Oil	68477-54-3 68516-20-1	
83618	Dicyclopentadiene Resin Grade (3a,4,7,7a-tetrahydo-4,7-methano-1H-indene/Alkenes, C9-11, C10-rich)	2647-00-4	Group III: MCPD Dimer
83985	Resin Feed (Distillates (petroleum), steam-cracked, C8-12 fraction/C9 mixture rich in indene and vinyltoluene/Complex mixture of (mainly aromatic) C9–C10 hydrocarbons); Dicyclopentadiene Resin	68477-54-3	Not Defined Properly
83879	Resin Distillates, steam cracked. C8-C12 (Extract residue (coal), light oil, alk, acid ext, indene fraction	68477-54-3	
83998	Resin—Distillate cracked, ethylene manufacturing by-product, C9-C10		

* Sample IDs and sponsored stream information as provided by the ACC Olefins Panel. ^#^ CAS RN and human health hazard subcategory assignments were made by the authors by matching the stream names, as provided by the sponsor, to the information in the US EPA Screening-Level Hazard Characterization documents for Low Benzene Naphthas, Resin Oils, and Cyclodiene Dimer Concentrates categories [26,27].

**Table 2:** Typical (as defined in [48]) versus observed (this study) constituents for substances in the Low Benzene Naphthas category.

Constituent	CAS RN	Formula	Typical Concentration (%)	Typical Concentration Range (%)	Observed (IMS-MS) Range (%)
Toluene	108-88-3	C7H8	~30	0–≤50	0–0.08
Ethylbenzene	100-41-4	C8H10	~20	0–≤45	0–0.2
Xylenes	1330-20-7	C8H10	~15	0–≤30	0–0.2
m-Xylene	108-38-3	C8H10	~7	0–≤15	0–0.2
p-Xylene	106-42-3	C8H10	~5	0–≤10	0–0.2
o-Xylene	95-47-6	C8H10	~2.5	0–≤5	0–0.2
Ethyltoluene	25550-14-5	C9H12	~20	0–≤45	0.01–0.21
1,2,4-Trimethylbenzene	95-63-6	C9H12	~12	0–≤21	0.01–0.21
Propylbenzene	103-65-1	C9H12	~8	0–≤15	0.01–0.21
1,2,3-Trimethylbenzene	526-73-8	C9H12	~6	0–≤12	0.01–0.21
Isopropylbenzene	98-82-8	C9H12	~1.5	0–≤9	0.01–0.21
3-Ethyltoluene	620-14-4	C9H12	~3	0–≤5	0.01–0.21
4-Ethyltoluene	622-96-8	C9H12	~1	0–≤2	0.01–0.21
1,3,5-Trimethylbenzene	108-67-8	C9H12	~1	0–≤2	0.01–0.21
Indene	95-13-6	C9H8	~15	0–≤40	0.22–2.6
Methylstyrene	1319-73-9	C9H10	~5	0–≤36	0.05–1.3
Indane	496-11-7	C9H10	~7	0–≤13	0.05–1.3
2,3,3a,4,7,7a-Hexahydro-4,7-methano-1H-indene	19398-83-5	C10H14	~22	0–≤30	0–0.49
Dihydrodicyclopentadiene	4488-57-7	C10H14	~15	0–≤25	0–0.5
1,2,3,5-Tetramethylbenzene	527-53-7	C10H14	~8	0–≤16	0–0.5
1,2,4,5-Tetramethylbenzene	95-93-2	C10H14	~6	0–≤11	0–0.5
1,2-Dimethyl-4-ethylbenzene	934-80-5	C10H14	~5	0–≤11	0–0.5
1,3-Dimethyl-4-ethylbenzene	874-41-9	C10H14	~2	0–≤4	0–0.5
1,4-Dimethyl-2-ethylbenzene	1758-88-9	C10H14	~2	0–≤3	0–0.5
2-Methyl-2-butene	513-35-9	C5H10	~7	0–≤14	0–1.32
Cyclopentane	287-92-3	C5H10	~6	0–≤11	0–1.32
Trans-2-pentene	646-04-8	C5H10	~5	0–≤10	0–1.32
Cis-2-pentene	627-20-3	C5H10	~2	0–≤3	0–1.32
Naphthalene	91-20-3	C10H8	~6	0–≤12	0.41–6.7
Tetralin	119-64-2	C10H12	~3	0–≤6	0.05–1.4
Dicyclopentadiene	77-73-6	C10H12	~15	0–≤2	0.05–1.4
Styrene	100-42-5	C8H8	~2	0–≤5	0.01–0.26
1-Methylnaphthalene	90-12-0	C11H10	~1	0–≤2	0.7–2.7
Isomer of Methylindene	N/A	N/A	~13	0–≤36	n.d.
C-10 Aromatic	N/A	N/A	~13	0–≤36	n.d.
N-pentane	109-66-0	C5H12	~16	0–≤31	n.d.
Isopentane	78-78-4	C5H12	~9	0–≤17	n.d.
Methylcyclohexane	108-87-2	C7H14	~14	0–≤27	n.d.
Ethylcyclopentane	1640-89-7	C7H14	~12	0–≤23	n.d.
Cis-1,2-dimethylcyclopentane	1192-18-3	C7H14	~2	0–≤3	n.d.
Cyclopentene	142-29-0	C5H8	~7	0–≤14	n.d.
N-heptane	142-82-5	C7H16	~7	0–≤14	n.d.
Tetrahydrodicyclopentadiene	6004-38-2	C10H16	~5	0–≤10	n.d.
N-octane	111-65-9	C8H18	~4	0–≤7	n.d.
Benzene	71-43-2	C6H6	~0	<0.1	n.d.

**Table 3:** Typical (as defined in [49]) versus observed (this study) constituents for substances in the Resin Oils category.

Constituent	CAS RN	Formula	Typical Concentration (%)	Typical Concentration Range (%)	Observed (IMS-MS) Range (%)
DCPD	77-73-6	C10H12	~40	0–≤80	0.02–0.55
Vinyltoluene	25013-15-4	C9H10	~30	0–≤60	0.03–0.37
4-Methylstyrene	622-97-9	C9H10	~20	0–≤40	0.03–0.37
Indan	496-11-7	C9H10	~7.5	0–≤25	0.03–0.37
2-Phenylpropene	98-83-9	C9H10	~5	0–≤20	0.03–0.37
3-Methylstyrene	100-80-1	C9H10	~10	0–≤20	0.03–0.37
2-Methylstyrene	611-15-4	C9H10	~7.5	0–≤15	0.03–0.37
Cyclopentane	287-92-3	C5H10	~25	0–≤50	0–0
2-Methylbut-2-ene	513-35-9	C5H10	~5	0–≤10	0–0
Ethyltoluene	25550-14-5	C9H12	~20	0–≤40	0–0.19
Trimethylbenzenes (TMB)	25551-13-7	C9H12	~20	0–≤40	0–0.19
Isopropylbenzene	98-82-8	C9H12	~15	0–≤30	0–0.19
1,2,4-Trimethylbenzene	95-63-6	C9H12	~7.5	0–≤15	0–0.19
m-Ethyltoluene	620-14-4	C9H12	~5	0–≤13	0–0.19
1,3,5-Trimethylbenzene	108-67-8	C9H12	~5	0–≤10	0–0.19
Propylbenzene	103-65-1	C9H12	~5	0–≤10	0–0.19
4,7-Methano-1H-indene, 2,3,3a,4,7,7a-hexahydro-	19398-83-5	C10H14	~10	0–≤20	0–0.2
Dihydrodicyclopentadiene	4488-57-7	C10H14	~5	0–≤12	0–0.2
1,2,4,5-Tetramethylbenzene	95-93-2	C10H14	~5	0–≤10	0–0.2
Xylenes	1330-20-7	C8H10	~10	0–≤20	0–0.11
Ethylbenzene	100-41-4	C8H10	~5	0–≤15	0–0.11
Naphthalene	91-20-3	C10H8	~20	0–≤40	0.13–3.14
Methylnaphthalene	90-12-0	C11H10	~5	0–≤15	0.08–2.04
Methyldicyclopentadiene	25321-13-5	C11H14	~10	0–≤21	0.02–0.44
Toluene	108-88-3	C7H8	~10	0–≤20	0–0.03
Styrene	100-42-5	C8H8	~12.5	0–≤25	0–0.04
Indene	95-13-6	C9H8	~35	0–≤80	0.16–1.67
4-Ethyl-3-octene	53966-51-1	C10H20	~40	0–<80	n.d.
Methylindenes	29036-25-7	C10H10	~10	0–≤70	n.d.
1,2-Dihydronaphthalene	447-53-0	C10H10	~12.5	0–≤25	n.d.
2,3,6-Trimethyl-4-octene	63830-65-9	C11H22	~20	0–≤50	n.d.
1,3-Pentadiene	504-60-9	C5H8	~16	0–≤51	n.d.
Cyclopentene	142-29-0	C5H8	~15	0–≤25	n.d.
(3Z)-Penta-1,3-diene	1574-41-0	C5H8	~10	0–≤20	n.d.
(E)-3-Dodecene	7239-23-8	C12H24	~5	0–≤10	n.d.
Benzene	71-43-2	C6H6	~1.0	0–≤3	n.d.
Phenol	108-95-2	C6H6O	~0	0–≤7	n.d.
n-Hexane	110-54-3	C6H14	~0	0–≤0.2	n.d.

## Data Availability

All pertinent data are included in Supplementary Materials.

## Supplementary Materials

The following are available online at https://www.mdpi.com/article/10.3390/toxics11070586/s1, Table S1: LBN & RO US EPA Human Health Hazard Subcategory Assignments; Table S2: Raw IMS-MS Data Matrix (Before Processing) for Neat Samples; Table S3: Raw IMS-MS Data Matrix (Before Processing) for DMSO Extracts; Table S4: Filtered IMS-MS Data Matrix (After Processing) for Neat Samples; Table S5: Filtered IMS-MS Data Matrix (After Processing) for DMSO Extracts; Table S6: Filtered IMS-MS Data Matrix with Molecular Formula Assignments: Neat Samples; Table S7: Filtered IMS-MS Data Matrix with Molecular Formula Assignments: DMSO Extracts; Table S8: Cell-Specific Phenotypes and Endpoints Measured; Table S9: Positive Controls for All Cell Types Tested; Table S10: Links to US EPA HPV Documents for LBN and RO Categories; Table S11: LBN: Detailed Analyses of Expected vs. Observed Constituent Abundances; Table S12: RO: Detailed Analyses of Expected vs. Observed Constituent Abundances; Table S13: Potential Identities for Features Driving Bioactivity.

## Abbreviations

UVCB, unknown, variable, complex reaction byproducts, or biological materials; HPV, high production volume; POD, point of departure; IMS-MS, ion mobility spectrometry-mass spectrometry; GC-MS, gas chromatography; PAC, polycyclic aromatic compounds; LBN, low benzene naphthas; RO, resin oils and cyclodiene dimer concentrates; DMSO, Dulbecco’s Modified Eagle Medium; HUVEC, human umbilical vein endothelial cells; iPSC, induced pluripotent stem cell; ACC, American Chemistry Council; CAS, Chemical Abstract Service; ASTM, American Society for Testing and Materials; QTOF, quadrupole time of flight; APPI, atmospheric pressure photoionization; CCS, collisional cross section; DT, drift time; KMD, Kendrick mass defect; DBE, double bond equivalence; ToxPi, Toxicological Prioritization Index; ECHA, European Chemicals Agency; REACH, Registration, Evaluation, and Authorization of Chemicals; DCPD, dicyclopentadiene; MCPD, methylcyclopentadiene dimer; FT-ICR, Fourier transform ion cyclotron resonance; MS, mass spectrometry.
