# Supplementary material for: Integrative Chemical–Biological Grouping of Complex High Production Volume Substances from Lower Olefin Manufacturing Streams
Source: Toxics. 2023 Jul 5;11(7):586. doi: 10.3390/toxics11070586 (PMC10385386; doi:10.3390/toxics11070586)
Supplement: Supplementary file 1 [file toxics-11-00586-s001.zip › toxics-2446194-SI/Supplemental Information Table of Contents.pdf]

Table of Contents: Supplemental Information

1. LBN & RO US EPA Human Health Hazard Subcategory Assignments
2. Raw IMS-MS Data Matrix (Before Processing) for Neat Samples
3. Raw IMS-MS Data Matrix (Before Processing) for DMSO Extracts
4. Filtered IMS-MS Data Matrix (After Processing) for Neat Samples
5. Filtered IMS-MS Data Matrix (After Processing) for DMSO Extracts
6. Filtered IMS-MS Data Matrix with Molecular Formula Assignments: Neat Samples
7. Filtered IMS-MS Data Matrix with Molecular Formula Assignments: DMSO Extracts
8. Cell-Specific Phenotypes and Endpoints Measured
9. Positive Controls for All Cell Types Tested
10. Links to US EPA HPV Documents for LBN and RO Categories
11. LBN: Detailed Analyses of Typical vs. Observed Constituent Abundances
12. RO: Detailed Analyses of Typical vs. Observed Constituent Abundances
13. Potential Identities for Features Driving Bioactivity
